# Evaluation of drug-induced lymphocyte stimulation test in mesalazine-associated allergic drug reaction

**DOI:** 10.1016/j.jacig.2025.100625

**Published:** 2025-12-17

**Authors:** Naoto Fukasawa, Hiroki Kiyohara, Takeya Adachi, Shinya Sugimoto, Yusuke Yoshimatsu, Soichiro Murakami, Ichiro Mizushima, Yuta Kaieda, Kaoru Takabayashi, Junya Tsunoda, Nobuhiro Nakamoto, Koichi Fukunaga, Masataka Taguri, Yohei Mikami, Takanori Kanai

**Affiliations:** a Division of Gastroenterology and Hepatology, Department of Internal Medicine, Keio University School of Medicine, Tokyo, Japan; b Department of Dermatology, Keio University School of Medicine, Tokyo, Japan; c Department of Surgery, Keio University School of Medicine, Tokyo, Japan; d Division of Pulmonary Medicine, Department of Internal Medicine, Keio University School of Medicine Tokyo, Japan; e Allergy Center, Keio University Hospital, Tokyo, Japan; f Department of Health Data Science, Tokyo Medical University, Tokyo, Japan

**Keywords:** 5-Aminosalicylic acid, drug-induced lymphocyte stimulation test, cross-reactivity, 5-ASA intolerance, ulcerative colitis, inflammatory bowel disease, lymphocyte transformation test

## Abstract

**Background:**

5-Aminosalicylic acid (5-ASA) is a fundamental drug for UC management; however, adverse reactions can lead to poor clinical outcomes and increased health care costs. No objective tests currently exist to predict adverse reactions. This study thus investigated 5-ASA across 4 formulations and addressed a key issue in the treatment of mild to moderate ulcerative colitis (UC).

**Objective:**

We aimed to examine the utility of the drug-induced lymphocyte stimulation test (DLST) in predicting successful rotation from mesalazine to sulfasalazine (SASP) in patients with UC.

**Methods:**

We retrospectively analyzed the largest cohort to date of patients with UC who were suspected of 5-ASA–associated adverse reactions and underwent DLST. We evaluated the DLST positivity rate for the suspected formulation, cross-reactivity to nonsuspected formulations, and clinical outcomes after rotation from mesalazine to SASP.

**Results:**

Mesalazine formulations exhibited a DLST positivity rate of 22.0% (18/82), with 45.1% (37/82) of patients testing positive for at least one 5-ASA formulation. Cross-reactivity between mesalazine and SASP was lower at 12.2% (10/82), likely because of structural differences. Adverse reactions typically developed within 2 weeks of initiating 5-ASA and commonly included fever, diarrhea, and bloody stool. Among patients with mesalazine-associated adverse reactions with DLST-negative test result for SASP, 8 of 12 tolerated the rotation. Rotation failure was mainly associated with a positive or borderline DLST result for mesalazine.

**Conclusions:**

DLST serves as a useful diagnostic tool for guiding treatment in patients with UC and suspected 5-ASA–associated hypersensitivity reactions. Prospective multicenter studies are needed to validate its clinical utility.

Ulcerative colitis (UC) is a chronic inflammatory bowel disease of unknown cause that affects the rectum and colon.^1^ UC is typically classified by disease severity (mild, moderate, or severe) and by location (proctitis, left-sided colitis, pancolitis, or right-sided colitis).^1^^,^^2^ Multiple factors, including genetic predisposition, environmental influences, gut microbiota, and immune dysregulation, are believed to contribute to its development. Recent insights into the underlying pathophysiology have led to the emergence of new treatment options.^3^, ^4^, ^5^ However, many of these newer therapies have potential adverse effects due to immunosuppression, are costly, and are generally reserved for moderate to severe or treatment-resistant cases.^6^ The first-line drug for induction therapy and maintenance therapy for patients with mild to moderate UC remains 5-aminosalicylic acid (5-ASA).^1^^,^^7^^,^^8^ 5-ASA is available in oral and topical forms; when monotherapy is ineffective, combination therapy with oral and topical 5-ASA is considered an effective approach.^9^

Although 5-ASA preparations are the primary treatment for mild to moderate UC, approximately 5% to 10% of patients experience adverse reactions such as fever, diarrhea, bloody stool, and abdominal pain, which can make continued treatment difficult.^10^, ^11^, ^12^ Among patients receiving anti–tumor necrosis factor biologic agents, 5-ASA–associated adverse reactions are associated with an increased need for additional induction therapy after the initiation of biologics.^13^ Therefore, preventing adverse events and avoiding increased health care costs related to advanced therapies is essential. Furthermore, patients with 5-ASA–associated adverse reactions have significantly higher rates of surgical intervention (11.5% vs 2.6%; hazard ratio = 4.92; 95% confidence interval, 2.58-9.38), which may worsen clinical outcomes.^14^ In clinical practice, the main 5-ASA formulations include sulfasalazine (SASP) and 3 mesalazine agents commonly used in Japan: time-dependent mesalazine (Pentasa), pH-dependent mesalazine (Asacol), and once-daily multimatrix system mesalazine (Lialda). SASP is metabolized by intestinal bacteria into mesalazine, which has anti-inflammatory effects, and sulfapyridine, which may cause adverse effects such as headache, dizziness, and fever. In contrast, mesalazine, developed as a modified version of SASP, contains only the active ingredient.^15^^,^^16^ The lower tolerability of SASP compared with mesalazine is likely due to this structural difference (risk ratio = 0.48; 95% confidence interval, 0.36-0.63).^15^

Despite the strong similarity between symptoms of 5-ASA–associated adverse reaction and those of primary UC exacerbation, no definitive or clinically practical test exists to confirm or exclude 5-ASA–associated adverse reactions. The drug-induced lymphocyte stimulation test (DLST), originally developed to diagnose type IV allergies, has also been used to detect 5-ASA–associated hypersensitivity reactions based on the hypothesis that T-cell reactivation contributes to allergic responses.^17^ DLST is considered a useful diagnostic adjunct because of its high specificity in identifying 5-ASA–associated hypersensitivity reactions; however, its clinical significance remains limited by low sensitivity, with a sensitivity of 0.24 and a specificity of 0.81.^18^ Desensitization therapy or rotation to an alternative 5-ASA formulation has been used to reduce the overuse of advanced therapies, including biologics and Janus kinase inhibitors, and has been reported in small-scale clinical studies.^10^^,^^11^ However, decisions to rotate formulations in patients whose disease can tolerate 5-ASA therapy have typically relied on shared decision-making between patients and physicians rather than objective clinical data. In clinical practice, we sometimes encounter cases in which rotation to SASP is effective for patients with mesalazine-associated adverse reactions; however, there are no reports demonstrating the potential cross-reactivity between 5-ASA formulations based on DLSTs results. Moreover, no clear strategy based on laboratory data has been clearly established.

Therefore, in this study, we aimed to determine the DLST positivity rate for each 5-ASA formulation and to assess cross-reactivity among formulations in patients with UC who underwent DLST because of suspected 5-ASA–associated adverse reactions. The goal was to facilitate patient stratification and guide successful rotation to SASP.

## Methods

### Patients

We conducted a single-center retrospective observational study using the Keio University Hospital information database. We reviewed the medical records of patients with UC who were diagnosed with 5-ASA–associated adverse reactions and underwent DLST (n = 74) between January 1, 2018, and April 21, 2025.

### Definition

5-ASA–associated adverse reactions were clinically defined as the discontinuation of 5-ASA therapy as a result of the onset of symptoms such as fever, diarrhea, bloody stool, and abdominal pain after treatment initiation.^11^^,^^12^^,^^19^ DLST was performed to evaluate lymphocyte reactivity to both suspected and nonsuspected 5-ASA formulations. We evaluated DLST positivity rate for the suspected formulation and assessed cross-reactivity with other, nonsuspected 5-ASA formulations. For example, if a patient developed adverse reactions after receiving mesalazine and subsequently discontinued it, then we determined the DLST positivity rate for mesalazine (eg, Pentasa) and evaluated the cross-reactivity with Asacol, Lialda, and SASP.

### Outcomes

We assessed the DLST positivity rate for each 5-ASA formulation and evaluated cross-reactivity among different 5-ASA agents. We recorded time from drug initiation to onset of adverse reactions, types of symptoms, and subsequent management strategies. In patients with 5-ASA–associated adverse reactions, we also evaluated rotation to SASP and its continuation rate to determine the feasibility and predictive value of DLST-guided rotation strategies.

### DLST

DLST (SRL, Tokyo, Japan) measures the proliferation of sensitized lymphocytes in response to a suspected drug by quantifying the uptake of tritiated thymidine (^3^H-thymidine). This test evaluates lymphocyte reactivity in patients suspected of having drug allergies. For each patient, 12 mL of blood was collected per tablet or capsule of the 5-ASA formulation and placed in a heparinized blood collection tube. An additional 5 mL of blood was collected for each extra tablet or capsule tested. The stimulation index was calculated as the ratio of ^3^H-thymidine uptake in the drug-containing mixture to that in the drug-free control. A stimulation index of >180% was considered DLST positive, while a stimulation index between 160% and 180% was considered DLST borderline.

### Ethical considerations

This study was approved by the ethics committee of Keio University School of Medicine (approval 20150100) and was conducted in accordance with the principles of the Declaration of Helsinki. Because informed consent was not required for this retrospective observational study, patient consent was obtained by publicly posting study information within Keio University Hospital. All patient records and data were anonymized before analysis.

### Statistical analysis

Statistical analysis was conducted by StataNow BE v19 software (StataCorp, College Station, Tex). The association between DLST results and outcomes of SASP rotation was assessed by the Fisher exact test. *P* < .05 was considered statistically significant. To evaluate cross-reactivity among 5-ASA preparations, we created Sankey diagrams using the SankeyMATIC website (sankeymatic.com).

## Results

### Patient characteristics

The DLST was performed in 82 patients with UC who were suspected of having experienced 5-ASA–associated adverse reactions (mean age, 39.8 ± 17.6 years; 41 men and 41 women). Among them, 78.4% underwent DLST with all four 5-ASA formulations. Patient characteristics are summarized in Table I. The suspected 5-ASA formulations associated with adverse reactions symptoms included oral Pentasa (n = 20), Pentasa suppositories (n = 3), Asacol (n = 22), Lialda (n = 30), and SASP (n = 7). The extent of colitis was pancolitis in 58.5% of patients, left-sided colitis in 20.7%, and proctitis in 12.2%.

**Table I:** Baseline characteristics

Characteristic	Mesalazine				SASP	5-ASA
	Pentasa oral	Pentasa suppository	Asacol	Lialda		
No. of patients	20	3	22	30	7	82
Age (years), median (IQR)	45.4 (19-73)	37.3 (19-59)	37.6 (15-64)	35.8 (17-77)	46.6 (21-77)	39.8 (15-77)
Sex						
Male	13 (65.0)	1 (33.3)	11 (50.0)	12 (40.0)	4 (57.1)	41 (50.0)
Female	7 (35.0)	2 (66.7)	11 (50.0)	18 (60.0)	3 (42.9)	41 (50.0)
Extent of colitis						
Pancolitis	10 (50.0)	1 (33.3)	14 (63.6)	18 (60.0)	5 (71.4)	48 (58.5)
Left-sided colitis	5 (25.0)	2 (66.7)	3 (13.6)	6 (20.0)	1 (14.3)	17 (20.7)
Proctitis	1 (5.0)	0	3 (13.6)	5 (16.7)	1 (14.3)	10 (12.2)
Right-sided or segmental colitis	4 (20.0)	0	2 (9.1)	1 (3.3)	0	7 (8.5)
Patients with 4 DLST	17 (85.0)	3 (100.0)	21 (95.5)	23 (76.7)	2 (28.6)	66 (80.5)

Data are presented as nos. (%) unless otherwise indicated.

### 5-ASA–associated adverse reactions and onset period

5-ASA–associated adverse reactions and time to symptom onset for each suspected drug are presented in Table II. In 43.9% of cases, symptoms developed within 2 weeks of starting 5-ASA therapy, while 32.9% experienced symptom onset at 4 weeks or later. The most frequently reported adverse reactions were consistent with previous findings,^10^^,^^12^ with fever being the most common (46.3%, n = 38), followed by worsening diarrhea (42.7%, n = 35) and worsening bloody stool (26.8%, n = 22). Additional symptoms included skin reactions (18.3%, n = 15), abdominal pain (15.9%, n = 13), nausea (6.1%, n = 5), pancreatitis (4.9%, n = 4), pneumonia (3.7%, n = 3), interstitial pneumonia (2.4%, n = 2), arthralgia (2.4%, n = 2), headache (2.4%, n = 2), myalgia (2.4%, n = 2), chest pain (1.2%, n = 1), fatigue (1.2%, n = 1), and liver dysfunction (1.2%, n = 1). Some patients experienced overlapping symptoms.

**Table II:** 5-ASA–associated adverse reactions and time to onset for each suspected drug

Characteristic	Mesalazine				SASP	5-ASA
	Pentasa oral	Pentasa suppository	Asacol	Lialda		
No. of subjects	20	3	22	30	7	82
Period from start of administration to onset of symptoms						
<1 week	3 (15.0)	1 (33.3)	2 (9.1)	8 (26.7)	1 (14.3)	15 (18.3)
1 week	2 (10.0)	0	4 (18.2)	3 (10.0)	1 (14.3)	10 (12.2)
2 weeks	4 (20.0)	0	4 (18.2)	2 (6.7)	1 (14.3)	11 (13.4)
3 weeks	0	0	1 (4.5)	0	0	1 (1.2)
≥4 weeks	6 (30.0)	1 (33.3)	5 (22.7)	11 (36.7)	4 (57.1)	27 (32.9)
Unknown	5 (25.0)	1 (33.3)	6 (27.3)	6 (20.0)	0	18 (22.0)
Adverse reactions						
Fever	12 (60.0)	1 (33.3)	7 (31.8)	16 (53.3)	2 (28.6)	38 (46.3)
Diarrhea	10 (50.0)	2 (66.7)	10 (45.5)	12 (40.0)	1 (14.3)	35 (42.7)
Blood in stool	3 (15.0)	0	10 (45.5)	7 (23.3)	2 (28.6)	22 (26.8)
Skin symptoms	1 (5.0)	0	4 (18.2)	5 (16.7)	5 (71.4)	15 (18.3)
Abdominal pain	4 (20.0)	0	6 (27.3)	3 (10.0)	0	13 (15.9)
Nausea	2 (10.0)	0	3 (13.6)	0	0	5 (6.1)
Pancreatitis	2 (10.0)	0	0	2 (6.7)	0	4 (4.9)
Pneumonia	1 (5.0)	0	1 (4.5)	1 (3.3)	0	3 (3.7)
Interstitial pneumonia	1 (5.0)	0	0	1 (3.3)	0	2 (2.4)
Arthralgia	0	0	0	2 (6.7)	0	2 (2.4)
Headache	0	0	0	2 (6.7)	0	2 (2.4)
Myalgia	1 (5.0)	0	1 (4.5)	0	0	2 (2.4)
Chest pain	0	0	0	1 (3.3)	0	1 (1.2)
Fatigue	0	0	1 (4.5)	0	0	1 (1.2)
Liver dysfunction	0	0	1 (4.5)	0	0	1 (1.2)

Data are presented as nos. (%) unless otherwise indicated.

### DLST results for 5-ASA formulations

The DLST positivity rates for each 5-ASA formulation are presented in Table III. The overall DLST positivity rate for the suspected formulation was 22.0%, with the highest rate observed for Pentasa suppository (33.3%). Patients with a positive DLST for the suspected drug presented less often with single-organ symptoms (25.9%) but more frequently with multiorgan or systemic symptoms (74.1%) compared with patients with a negative DLST (see Table E1 in this article’s Online Repository available at www.jaci-global.org). Regarding potential cross-reactivity, the DLST positivity rate was 32.9% for mesalazine formulations other than the suspected one, 12.2% for SASP, and 45.1% for at least one of the tested 5-ASA formulations. These findings indicate that cross-reactivity among mesalazine formulations is relatively common, whereas cross-reactivity with SASP is less frequent.

**Table III:** DLST positivity rate for each suspected drug

		Suspected 5-ASA formulation				
Characteristic	All 5-ASA	Mesalazine				SASP
		Pentasa oral	Pentasa suppository	Asacol	Lialda	
No. of subjects	82	20	3	22	30	7
Suspected drug						
Positive	18 (22.0)	4 (20.0)	1 (33.3)	7 (31.8)	6 (20.0)	0
Positive + borderline	27 (32.9)	7 (35.0)	2 (66.7)	8 (36.4)	10 (33.3)	0
Cross-reactivity to mesalazine (excluding suspected formulation)						
Positive	27 (32.9)	10 (50.0)	0	9 (40.9)	6 (20.0)	2 (28.6)
Positive + borderline	37 (45.1)	10 (50.0)	2 (66.7)	12 (54.5)	11 (36.7)	2 (28.6)
Cross-reactivity to SASP						
Positive	10 (12.2)	5 (25.0)	1 (33.3)	2 (9.1)	2 (6.7)	…
Positive + borderline	15 (18.3)	7 (35.0)	1 (33.3)	3 (13.6)	4 (13.3)	…
At least one of all tested 5-ASA agents						
Positive	37 (45.1)	11 (55.0)	2 (66.7)	11 (50.0)	11 (36.7)	2 (28.6)
Positive + borderline	48 (58.5)	13 (65.0)	2 (66.7)	13 (59.1)	18 (60.0)	2 (28.6)

Data are presented as nos. (%) unless otherwise indicated.

### Continuation rate of rotation to SASP in patients with mesalazine-associated adverse reactions

**Fig 1:**
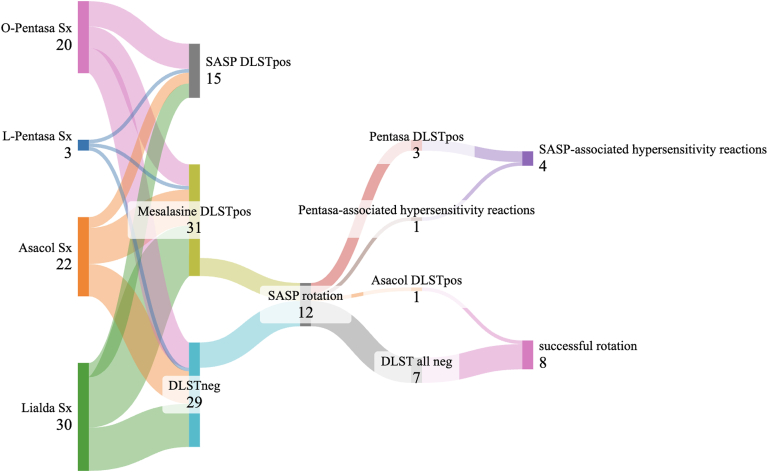
Evaluation of DLST cross-reactivity in cases of SASP rotation by Sankey diagrams. DLST cross-reactivity for all 5-ASA formulations is shown, including cases in which SASP rotation was performed in mesalazine-positive, SASP-negative group, and in group negative for all 5-ASA formulations. SASP rotation was performed in 12 cases, and rotation was successful in 8 cases. Four cases of adverse reactions to SASP rotation in this analysis were either Pentasa DLST positive or occurred in patients clinically suspected of having adverse reactions to Pentasa. *Asacol DLSTpos,* DLST positive for Asacol in patients with SASP rotation; *Asacol Sx,* cases of Asacol as suspected drug causing adverse reactions; *DLST all neg,* DLST negative for all 5-ASA formulations in patients with SASP rotation; *DLSTneg,* DLST negative for all 5-ASA formulations; *Lialda Sx,* cases of Lialda as suspected drug causing adverse reactions; *L-Pentasa Sx,* cases of Pentasa suppository as suspected drug causing adverse reactions; *mesalazine DLSTpos,* DLST negative for SASP and DLST positive for mesalazine; *O-Pentasa Sx,* cases of oral Pentasa as suspected drug causing adverse reactions; *Pentasa DLSTpos,* DLST positive for Pentasa in patients with SASP rotation; *Pentasa-associated adverse reactions,* Pentasa as suspected drug causing adverse reactions in patients with SASP rotation; *SASP DLSTpos,* DLST positive for SASP.

To assess the feasibility of switching to SASP in patients with mesalazine-associated adverse reactions, we generated a Sankey diagram, shown in Fig 1. Among 75 patients with mesalazine-associated adverse reactions, 15 tested positive for SASP on DLST, while 60 tested negative. Of the DLST-negative group, 31 were negative for SASP but positive for at least one mesalazine, and 29 cases were negative for all four 5-ASA agents. Among those who tested DLST negative for SASP, 12 underwent rotation to SASP after discontinuation of the suspected formulation.

Details of the DLST results and SASP continuation rates in patients who underwent SASP rotation are presented in Table IV. SASP was administered to 12 patients, 11 of whom had completed DLST testing for all four 5-ASA formulations. Of these, SASP rotation was successful in 8 patients, including 2 who required escalation to biologic therapy because of SASP’s insufficient efficacy and disease flare at 1 and 19 weeks after rotation, respectively. However, after SASP administration, neither of these patients developed SASP-associated hypersensitivity reactions such as rash; nor did they experience any acute worsening of gastrointestinal symptoms.

**Table IV:** SASP continuation rate and DLST positivity rate in patients with mesalazine-associated adverse reactions who underwent rotation to SASP

Patient no.	Suspected drug	Sex	Age (years)	Extent of colitis	Pentasa		Asacol		Lialda		SASP		Results of SASP rotation
					DLST	SI	DLST	SI	DLST	SI	DLST	SI	
1	Asacol	M	15	Proctitis	+/−	1.7	+/−	1.7	+	2.7	−	1.2	SASP-associated adverse reactions
2	Pentasa oral	M	71	Pancolitis	+/−	1.7	Untested	…	Untested	…	Untested	…	SASP-associated adverse reactions
3	Asacol	M	64	Left-sided colitis	+	3.4	−	1.4	−	1.5	−	1.1	SASP-associated adverse reactions
4	Pentasa oral	F	21	Left-sided colitis	−	1.4	−	1.5	+	2.1	−	1.0	SASP-associated adverse reactions
5	Lialda	F	19	Left-sided colitis	−	1.4	−	1.4	−	1.4	−	1.2	Treatment resistance
6	Lialda	F	26	Pancolitis	−	0.7	−	0.9	−	1.4	−	1.0	Treatment resistance
7	Asacol	F	52	Pancolitis	−	1.5	−	1.2	−	1.3	−	1.4	Continuous administration
8	Asacol	F	45	Right-sided or segmental colitis	−	1.5	−	0.8	−	1.2	−	1.4	Continuous administration
9	Lialda	F	24	Pancolitis	−	1.5	−	1.1	−	1.5	−	1.1	Continuous administration
10	Lialda	F	17	Proctitis	−	1.0	−	1.2	−	1.2	−	1.1	Continuous administration
11	Lialda	F	38	Left-sided colitis	−	1.4	+	2.0	−	1.4	−	1.4	Continuous administration
12	Pentasa suppository	M	19	Left-sided colitis	−	1.0	−	1.2	−	1.1	−	1.1	Continuous administration

*SI,* Stimulation index.

In contrast, 4 patients discontinued SASP because of adverse reactions. All were either DLST positive or borderline for Pentasa or had a clinical history suggestive of Pentasa-associated adverse reactions. Among data of the 11 patients who underwent DLST for all 4 formulations, 7 patients were negative for all agents, and all 7 successfully tolerated SASP. The Fisher exact test revealed a significant association between being DLST negative for the all four 5-ASA formulations and successful SASP rotation (*P* = .024).

## Discussion

In this study, we aimed to determine the positivity rate and cross-reactivity of the DLST among 5-ASA formulations in patients with UC and suspected 5-ASA–associated adverse reactions. Our findings demonstrate a relatively high DLST positivity rate for mesalazine formulations and significantly lower cross-reactivity with SASP. These results highlight DLST as a valuable tool for identifying patients whose disease permits safe rotation from mesalazine to SASP.

In our cohort, adverse reactions developed predominantly within 2 weeks of initiating 5-ASA therapy, with fever, diarrhea, and worsening bloody stool as the most common symptoms, consistent with previous reports.^10^^,^^12^ A key clinical challenge is distinguishing between 5-ASA–associated adverse reactions and UC exacerbation; both may present with overlapping symptoms such as worsening diarrhea and bloody stool, thus emphasizing the need for reliable diagnostic tools such as DLST to guide therapeutic decisions.

However, the DLST positivity rate for the suspected 5-ASA formulations was 22.0%, aligning with previous literature,^18^ which highlights DLST’s limited sensitivity but relatively high specificity as a method for detecting type IV drug allergies. Notably, cross-reactivity among mesalazine formulations was substantial (32.9% for cross-reactivity and 45.1% for at least one tested 5-ASA formulation), reinforcing clinical observations that adverse reactions to one mesalazine agent often predict adverse reactions to others. Conversely, the DLST positivity for SASP among patients with mesalazine-associated adverse reactions was lower (12.2%), supporting the hypothesis that structural differences between SASP and mesalazine may contribute to distinct immunologic responses. This difference might be attributable to the chemical structural differences between mesalazine and SASP.^16^^,^^20^

5-ASA–associated adverse reactions—often labeled in clinical practice, albeit imprecisely, as *5-ASA intolerance*—comprise two distinct categories: (1) immunologic drug hypersensitivity and (2) nonimmunologic adverse reactions. In our study, patients positive via DLST to the suspect drug more often exhibited multiorgan or systemic symptoms, supporting an underlying T-cell–mediated mechanism. Although the possibility of falsely negative DLST results precludes a definitive distinction, mesalazine-associated adverse reactions may be categorized into nonimmunologic adverse reactions and immunologic hypersensitivity. A positive mesalazine DLST result may indicate an immunologically mediated hypersensitivity reactions and should be regarded as a potential risk factor when attempting formulation rotation. In addition, single-organ symptoms (61.9%) predominated in the group with positive DLST to nonsuspected agents, suggesting that cross-reactivity may still give rise to clinical symptoms, even without systemic symptoms.

The practical implications of our findings are underscored by the successful rotation to SASP in 8 of 12 patients exhibiting mesalazine-associated adverse reactions but DLST negative for SASP. Notably, patients whose disease failed to respond to the SASP rotation were either DLST positive or borderline for Pentasa or had clinical histories strongly suggestive of Pentasa-associated adverse reactions. This highlights the need to consider both DLST results and clinical history—especially multiorgan or systemic symptoms—when making therapeutic decisions. Because adverse reactions to pH-dependent mesalazine (Asacol) and multimatrix system mesalazine (Lialda) may result from sensitivity to either the active ingredient (mesalazine) or an excipient, one patient with successful rotation to SASP from mesalazine likely experienced a reaction to an excipient rather than mesalazine itself. This supports the feasibility of SASP rotation in selected cases, enabling continued receipt of 5-ASA therapy, potentially reducing the need for escalation to immunosuppressive or biologic treatments, and lowering overall health care costs.

This study had some limitations, including its retrospective, single-center design, the possibility of selection bias, and a relatively small sample size. In addition, distinguishing adverse drug reactions from disease activity is inherently difficult, particularly in a retrospective study. Prospective multicenter studies are warranted to further validate the clinical utility of DLST in guiding treatment decisions for patients with suspected 5-ASA–associated hypersensitivity reactions.

In conclusion, our study evaluated the risk of adverse reactions’ recurrence after rotation to SASP in patients who had experienced mesalazine-associated adverse reactions, and we found that negative DLST results for all 4 agents may be associated with a lower risk of adverse reactions after SASP rotation. This approach has the potential to minimize the need for therapeutic escalation, lower health care costs, and improve patient outcomes.

**Clinical implication**

DLST helps identify UC patients with 5-ASA–associated hypersensitivity reactions who may successfully be rotated from mesalazine therapy to SASP, thereby minimizing therapeutic escalation, lowering health care costs, and improving clinical outcomes.

## Disclosure statement

Supported by the Advanced Research and Development Programs for Medical Innovation (AMED-CREST; JP21gm1510002 to T.K.); the Japan Society for the Promotion of Science (JSPS) KAKENHI (A) (JP24H00633 to Y.M. and JP20H00536 to T.K.); a JSPS Grant-in-Aid for Transformative Research Areas (B) (JP21H05123 to Y.M.); a Grant-in-Aid for Challenging Research (JP24K2212 to Y.M.); a Grant-in-Aid for Early-Career Scientists (JP22K16005 to H.K.); the Japan Science and Technology Agency FOREST (JPMJFR235H to Y.M.); the Ono Pharma Oncology, Immunology, Neurology Research Foundation; and the Keio University Medical Fund.

Disclosure of potential conflict of interest: T. Kanai received joint research support from Miyairisan Pharma, Mitsubishi Tanabe Pharma, Takeda Pharmaceutical, KYORIN Pharmaceutical, ZERIA Pharmaceutical, Daiichi Sankyo, and Mochida Pharmaceutical; lecture fees from Miyairisan Pharma and Takeda Pharmaceutical; endowed chair from EA Pharma, Kyorin Pharmaceutical, ZERIA Pharmaceutical, Mitsubishi Tanabe Pharma, JIMRO, and Mochida Pharmaceutical Miyarisan Pharma. H. Kiyohara received lecture fees from Mochida Pharmaceutical. The rest of the authors declare that they have no relevant conflicts of interest.
