# Supplementary material for: Evaluation of drug-induced lymphocyte stimulation test in mesalazine-associated allergic drug reaction
Source: J Allergy Clin Immunol Glob. 2025 Dec 17;5(2):100625. doi: 10.1016/j.jacig.2025.100625 (PMC12814058; doi:10.1016/j.jacig.2025.100625)
Supplement: Supplementary Table [file mmc1.docx]

Supplemental Table 1.

|  | Single-organ symptoms | | Multi-organ or systemic symptoms | | Cases (n) |
| --- | --- | --- | --- | --- | --- |
|  | n | [%] | n | [%] |  |
| Positive DLST for the suspected drug | 7 | 25.9 | 20 | 74.1 | 27 |
| Negative DLST for the suspected drug but positive for another drug | 13 | 61.9 | 8 | 38.1 | 21 |
| Negative DLST for all four drugs | 13 | 38.2 | 21 | 61.8 | 34 |
